# The prognostic value of the previous nephrectomy in pretreated metastatic renal cell carcinoma receiving immunotherapy: a sub-analysis of the Meet-URO 15 study

**DOI:** 10.1186/s12967-022-03601-6

**Published:** 2022-09-30

**Authors:** Sara Elena Rebuzzi, Alessio Signori, Giuseppe Luigi Banna, Annalice Gandini, Giuseppe Fornarini, Alessandra Damassi, Marco Maruzzo, Ugo De Giorgi, Umberto Basso, Silvia Chiellino, Luca Galli, Paolo Andrea Zucali, Emanuela Fantinel, Emanuele Naglieri, Giuseppe Procopio, Michele Milella, Francesco Boccardo, Lucia Fratino, Stefania Pipitone, Riccardo Ricotta, Stefano Panni, Veronica Mollica, Mariella Sorarù, Matteo Santoni, Alessio Cortellini, Veronica Prati, Hector Josè Soto Parra, Daniele Santini, Francesco Atzori, Marilena Di Napoli, Orazio Caffo, Marco Messina, Franco Morelli, Giuseppe Prati, Franco Nolè, Francesca Vignani, Alessia Cavo, Giandomenico Roviello, Pasquale Rescigno, Sebastiano Buti

**Affiliations:** 1 Medical Oncology Unit, Ospedale San Paolo, Savona, Italy; 2 Department of Internal Medicine and Medical Specialties (Di.M.I.), University of Genova, Genoa, Italy; 3 Department of Health Sciences, Section of Biostatistics, University of Genova, Genoa, Italy; 4 Candiolo Cancer Institute, FPO-IRCCS, Candiolo, Turin Italy; 5 Department of Oncology, Portsmouth Hospitals University NHS Trust, Portsmouth, UK; 6 Medical Oncology Unit 1, IRCCS Ospedale Policlinico San Martino, Genoa, Italy; 7 Oncology 1 Unit, Istituto Oncologico Veneto IOV - IRCCS, Padua, Italy; 8 Department of Medical Oncology, IRCCS Istituto Romagnolo per lo Studio dei Tumori (IRST) “Dino Amadori”, Meldola, Italy; 9 Medical Oncology Unit, IRCCS Policlinico San Matteo, Pavia, Italy; 10 Medical Oncology Unit 2, Azienda Ospedaliera Universitaria Pisana, Pisa, Italy; 11 Department of Biomedical Sciences, Humanitas University, Pieve Emanuele, Milan, Italy; 12 Department of Oncology, IRCCS, Humanitas Clinical and Research Center, Rozzano, Milano Italy; 13 Medical Oncology Unit, AUSL-IRCCS of Reggio Emilia, Reggio Emilia, Italy; 14 Department of Oncology, Azienda Ospedaliera Universitaria Integrata di Verona, University of Verona, Verona, Italy; 15 Division of Medical Oncology, IRCCS Istituto Tumori “Giovanni Paolo II”, Bari, Italy; 16 SS Oncologia Medica Genitourinaria, Fondazione IRCCS Istituto Nazionale dei Tumori, Milan, Italy; 17 Medical Oncology Unit, ASST - Istituti Ospitalieri Cremona Hospital, Cremona, Italy; 18 Department of Medical Oncology, Centro di Riferimento, Oncologico di Aviano CRO-IRCCS, Aviano, Italy; 19 Medical Oncology Unit, Department of Oncology and Hemathology, University Hospital of Modena, Modena, Italy; 20 Oncology Unit, Istituto di Ricovero e Cura a Carattere Scientifico (IRCCS) MultiMedica, Milan, Italy; 21 Medical Oncology, IRCCS Azienda Ospedaliero-Universitaria di Bologna, Bologna, Italy; 22 Department of Experimental, Diagnostic and Specialty Medicine, S.Orsola-Malpighi University Hospital, University of Bologna, Bologna, Italy; 23 U.O. Oncologia, Ospedale di Camposampiero, Camposampiero, Italy; 24 Oncology Unit, Macerata Hospital, Macerata, Italy; 25 Medical Oncology Department, Campus Bio-Medico University of Rome, 00128 Rome, Italy; 26 Department of Surgery and Cancer, Imperial College London, Faculty of Medicine, Hammersmith Hospital, London, UK; 27 Department of Medical Oncology, Ospedale Michele e Pietro Ferrero, Verduno, (CN) ASL CN2 Italy; 28 Department of Oncology, Medical Oncology, University Hospital Policlinico-San Marco, Catania, Italy; 29 UOC Oncologia Medica, “Sapienza University”, Polo Pontino, Rome, Italy; 30 Medical Oncology Department, University Hospital, University of Cagliari, Cagliari, Italy; 31 Department of Urology and Gynecology, Istituto Nazionale Tumori IRCCS Fondazione G. Pascale, Naples, Italy; 32 Medical Oncology Department, Santa Chiara Hospital, Trento, Italy; 33 UOC Oncologia Medica, Istituto Fondazione G. Giglio, Cefalù, Italy; 34 Oncology Department, Gemelli Molise, Campobasso, Italy; 35 Department of Oncology and advanced technologies, AUSL - IRCCS Reggio Emilia, Reggio Emilia, Italy; 36 Medical Oncology Division of Urogenital & Head & Neck Tumors, IEO, European Institute of Oncology IRCCS, Milan, Italy; 37 Division of Medical Oncology, Ordine Mauriziano Hospital, Turin, Italy; 38 Oncology Unit, Villa Scassi Hospital, Genoa, Italy; 39 Department of Health Sciences, Section of Clinical Pharmacology and Oncology, University of Firenze, Florence, Italy; 40 Medical Oncology Unit, University Hospital of Parma, Parma, Italy; 41 Medicine and Surgery Department, University of Parma, Parma, Italy

**Keywords:** Metastatic renal cell carcinoma, Nephrectomy, Immunotherapy, Nivolumab, Prognostic, Meet URO score, Neutrophil to lymphocyte ratio, IMDC score, Bone metastases

## Abstract

**Background:**

Nephrectomy is considered the backbone of managing patients with localized and selected metastatic renal cell carcinoma (mRCC). The prognostic role of nephrectomy has been widely investigated with cytokines and targeted therapy, but it is still unclear in the immunotherapy era.

**Methods:**

We investigated the Meet-URO-15 study dataset of 571 pretreated mRCC patients receiving nivolumab as second or further lines about the prognostic role of the previous nephrectomy (received in either the localized or metastatic setting) in the overall population and according to the Meet-URO score groups.

**Results:**

Patients who underwent nephrectomy showed a significantly reduced risk of death (HR 0.44, 95% CI 0.32–0.60, *p* < 0.001) with a longer median overall survival (OS) (35.9 months vs 12.1 months), 1-year OS of 71.6% vs 50.5% and 2-years OS of 56.5% vs 22.0% compared to those who did not. No significant interaction between nephrectomy and the overall five Meet-URO score risk groups was observed (*p* = 0.17). It was statistically significant when merging group 1 with 2 and 3 and group 4 with 5 (*p* = 0.038) and associated with a longer OS for the first three prognostic groups (*p* < 0.001), but not for groups 4 and 5 (*p* = 0.54).

**Conclusions:**

Our study suggests an overall positive impact of the previous nephrectomy on the outcome of pretreated mRCC patients receiving immunotherapy. The clinical relevance of cytoreductive nephrectomy, optimal timing and patient selection deserves further investigation, especially for patients with Meet-URO scores of 1 to 3, who are the once deriving benefit in our analyses. However, that benefit is not evident for IMDC poor-risk patients (including the Meet-URO score groups 4 and 5) and a subgroup of IMDC intermediate-risk patients defined as group 4 by the Meet-URO score.

**Supplementary Information:**

The online version contains supplementary material available at 10.1186/s12967-022-03601-6.

## Introduction

Immune checkpoint inhibitors (ICIs) have drastically changed the treatment landscape of metastatic renal cell cancer (mRCC) in recent years [1, 2]. Based on the outcomes of the CheckMate-025 study, nivolumab became the first ICI approved for mRCC patients pretreated with vascular endothelial growth factor receptors (VEGFR) tyrosine kinase inhibitors (TKI) in 2015 [3]. Subsequently, many different ICI-based combinations have been approved in the first-line setting [4].

Despite their efficacy, not all mRCC patients achieve a long-term benefit from immunotherapies, and prognostic or predictive factors have not been well defined yet [5]. Recently, the multicentric retrospective Meet-URO 15 study investigated baseline peripheral blood inflammatory indices, alongside other clinical factors, as prognostic factors in 571 mRCC patients receiving nivolumab in the ≥ 2nd line setting [6]. A novel prognostic score was then developed, namely the Meet-URO score, by adding the neutrophil-to-lymphocyte ratio (NLR) and presence of bone metastases to the International Metastatic Renal Cell Carcinoma Database Consortium (IMDC) score. The Meet-URO score showed higher prognostic accuracy than the IMDC alone [6]. It was then externally validated in both mRCC patients treated with 2nd and 3rd line cabozantinib and those who received 1st line nivolumab plus ipilimumab combination [7, 8].

Radical or partial nephrectomy for the localized RCC and cytoreductive nephrectomy (CN) for selected mRCC are considered the backbone of managing kidney cancer patients [1, 2].

For decades, CN has been the standard of care in the upfront management of mRCC with cytokines and targeted therapy based on observational analyses and randomized prospective trials [9–12].

Two prospective randomized trials CARMENA [13] and SURTIME [14] challenged the value and timing of CN in patients with synchronous metastatic renal cell carcinoma receiving sunitinib highlighting that selection based on prognostic factors is critical [15]. Therefore, CN should be offered to selected patients defined by prognostic features according to the IMDC and Memorial Sloan Kettering Cancer Centre (MSKCC) criteria, performance status, tumor burden and metastatic sites [16–18]. However, the prognostic role of the previous nephrectomy, observed with cytokines and targeted therapy, is still controversial in mRCC patients receiving immunotherapy [19].

In the current analysis, we explored the prognostic impact of the previous nephrectomy in the overall population of a large retrospective study on pretreated mRCC patients receiving nivolumab and after patients stratification by the Meet-URO score.

## Methods

The Meet-URO 15 study was a multicentric retrospective analysis of 571 pretreated mRCC patients receiving nivolumab as a second or further treatment line. For the present analysis, we included patients with available data on the nephrectomy and Meet-URO score [6]. Patients’ characteristics were presented using absolute frequency and percentage for categorical variables, median and ranges for quantitative ones.

OS was the reference outcome calculated using the Kaplan–Meier (KM) method. Univariable and multivariable Cox regression analyses were performed to assess the association between the nephrectomy and OS. The multivariable model was adjusted for NLR, IMDC and bone metastases.

The interaction between the nephrectomy and Meet-URO score was assessed by the likelihood-ratio (LR) test aiming at investigating if the association of the nephrectomy with OS was different among the Meet-URO risk categories [6].

Results were reported as hazard ratios (HR) with their 95% confidence interval (95% CI). The statistical significance level was set at 0.05. All statistical analyses were performed using the software Stata v.16 (StataCorp 2019).

## Results

### Patients’ characteristics

Among the patients enrolled in the Meet-URO 15 study, nephrectomy and Meet-URO score data was available for 556/571 patients (97%). Patients’ characteristics are summarised in Table 1.

**Table 1:** Patients’ characteristics

	All patients (N = 556)	Nephrectomy (N = 490)	No-nephrectomy (N = 66)	*p* value
Gender				
Male	391 (70.3)	347 (70.8)	44 (66.7)	
Female	165 (29.7)	143 (29.2)	22 (33.3)	0.49
Age (median, range)	63 (18–85)	62 (18–85)	66 (40–84)	0.004
Histology				
Clear cell	464 (84.1)	407 (83.4)	57 (89.1)	
Non clear cell	88 (15.9)	81 (16.6)	7 (10.9)	0.34
Treatment line				
2nd line	384 (69.1)	333 (68.0)	51 (77.3)	
3rd line	118 (21.2)	106 (21.6)	12 (18.2)	
> 3rd line	54 (9.7)	51 (10.4)	3 (4.5)	0.21
NLR (median, IQR)	2.8 (1.9–4.3)	2.8 (1.9–4.0)	3.7 (2.5–5.0)	
< 3.2	331 (59.5)	306 (62.5)	25 (37.8)	< 0.001
≥ 3.2	225 (40.5)	184 (37.6)	41 (62.1)	
Bone metastases				
Yes	361 (64.9)	331 (67.8)	30 (45.5)	0.001
No	195 (35.1)	159 (32.5)	36 (54.5)	
IMDC score				
Favourable	129 (23.2)	127 (25.9)	2 (3.0)	< 0.001
Intermediate	358 (64.4)	312 (63.7)	46 (69.7)	
Poor	69 (12.4)	51 (10.4)	18 (27.3)	
Meet-URO score				
1	86 (15.5)	84 (17.1)	2 (3.1)	< 0.001
2	193 (34.7)	184 (37.6)	9 (13.6)	
3	153 (27.5)	129 (26.3)	24 (36.4)	
4	97 (17.5)	77 (15.7)	20 (30.3)	
5	27 (4.9)	16 (3.3)	11 (16.7)	

*NLR* neutrophil to lymphocyte ratio, *IMDC* International Metastatic *RCC* Database Consortium, *IQR* interquartile range

The majority of patients (490/556, 88%) had a previous nephrectomy, received nivolumab as 2nd line therapy (384/556, 69.1%) and were at intermediate-risk according to IMDC score (358/556, 64.4%).

At disease onset, IMDC classification was available for 498 of the 556 patients: 165 (33%) were favorable, 293 (59%) intermediate and 40 (8%) poor risk. The stratification of patients according to IMDC score at disease onset and nivolumab treatment start, and by IMDC and Meet-URO scores at nivolumab treatment start, are provided in Additional file 1: Fig. S1 and Additional file 2: Fig S2.

Of the 490 patients who underwent nephrectomy, 164 (33%) had synchronous metastases at disease onset, while the remaining 326 (67%) had a radical nephrectomy.

### Survival outcomes

At the time of data cut-off (July 2020), with a median follow-up of 16.3 months, 72.3% of patients experienced progressive disease (PD), and 46.2% died. The median OS (mOS) was 29.5 months (95% CI 22.7–45.6), and median progression-free survival (mPFS) 7.3 months (95% CI 5.8–9.1).

### Nephrectomy vs no-nephrectomy

Patients who had previous nephrectomy (n = 490) were younger (median age: 62 vs 66 years, p = 0.004), had a higher percentage of low NLR (< 3.2, p < 0.001), absence of bone metastases (p = 0.001) and favorable IMDC score (p < 0.001) compared to those who did not (n = 66) (Table 1). The number of patients who had nephrectomy progressively reduced from the first to the fifth Meet-URO score group (p < 0.001) (Tables 1 and 2).

**Table 2:** Distribution of patients who have undergone or not nephrectomy across the Meet-URO groups

Meet-URO score [2]	Nephrectomy (%)	No-nephrectomy (%)
1	98	2
2	95	5
3	84	16
4	79	21
5	59	41

**Fig. 1:**
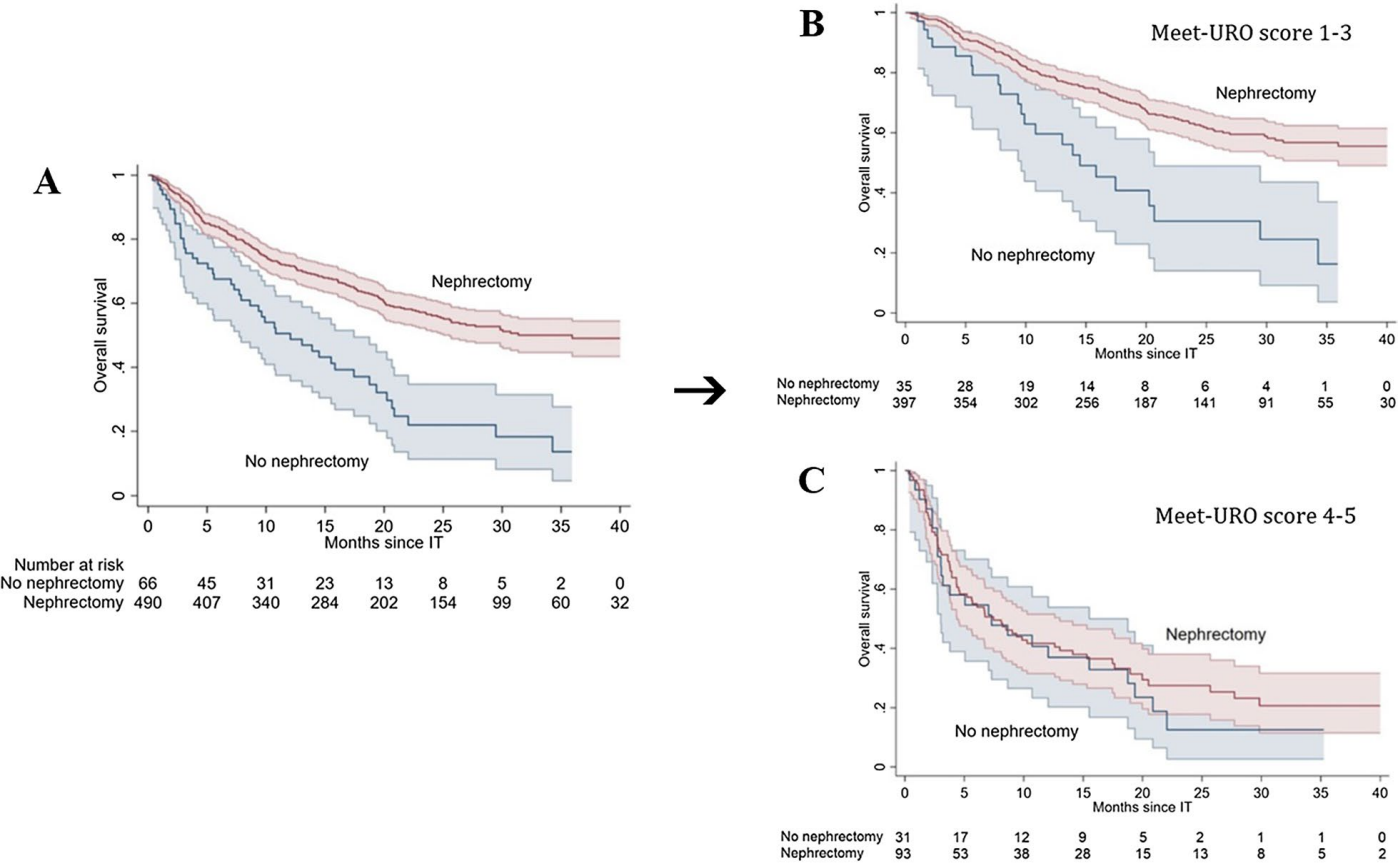
Kaplan Meiers curves showing the prognostic role of nephrectomy in mRCC patients: in the overall population (**A**), patients with Meet-URO scores 1,2,3 (**B**) and 4,5 (**C**).

Patients who had previous nephrectomy showed a significantly reduced risk of death (HR 0.44, 95% CI 0.32–0.60, *p* < 0.001) with a longer mOS (35.9 months, 95% CI 25.6–46.9 vs 12.1 months, 95% CI 7.7–17.4), 1-year OS of 71.6% (95% CI 67.3–75.4) vs 50.5% (95% CI 37.5–62.2) and 2-year OS of 56.5% (95% CI 51.5–61.1) vs 22.0% (95% CI 11.4–34.7) compared to those who did not (Fig. 1A).

The reduced risk of death for patients who had undergone nephrectomy was confirmed at the multivariable analysis (HR 0.70, 95% CI 0.50–0.99; *p* = 0.041) adjusted for NLR, IMDC and bone metastases.

When the presence of metastases at disease onset was considered, reduced risk of death by the previous nephrectomy was observed at the univariable analysis in both the metastatic (HR 0.48, 95% CI 0.33–0.69; *p* < 0.001) and non-metastatic (HR 0.40, 95% CI 0.28–0.56; *p* < 0.001) groups. At the multivariable analysis, the role of the previous nephrectomy was significantly confirmed only for patients with metastases at disease onset (HR 0.65, 95% CI 0.44–0.95; *p* = 0.025), whilst it did not reach the statistical significance in those without (HR 0.75, 95% CI 0.52–1.07; *p* = 0.11). (Additional file 3: Fig. S3).

### Correlation between nephrectomy and the Meet-URO score

Considering the original five Meet-URO score risk groups, we were not able to detect a significant interaction with nephrectomy (*p* = 0.17). Conversely, when merging group 1 with 2 and 3 and group 4 with 5 a significant interaction was observed (*p* = 0.038) and associated with a longer OS for the first three prognostic groups (*p* < 0.001), but not for groups 4 and 5 (*p* = 0.54) (Table 3, Fig. 1B, C).

**Table 3:** Interaction between the Meet-URO score and the prognostic role of nephrectomy

Meet-URO score	HR (95%CI) Nephrectomy vs No nephrectomy	*p* value for interaction	HR (95%CI) Nephrectomy vs No nephrectomy	*p* value for interaction
1	NE	*p* = 0.17	0.40 (0.25–0.63) *p* < 0.001	0.038
2	0.59 (0.23–1.47)			
3	0.45 (0.26–0.77)			
4	0.96 (0.53–1.73)		0.86 (0.54–1.38); *p* = 0.54	
5	1.00 (0.45–2.24)			

*HR* hazard ratio, *CI* confidence interval, *NE* Not estimable

## Discussion

Overall, nephrectomy could be beneficial as resectioning the primary tumor might eliminate the ‘immunological sink’, thus reducing the level of immunosuppressive cytokines and potentiating the anti-tumor immune response [20]. In this context, nephrectomy might be even more relevant for patients who receive ICI for metastatic disease [19].

Moreover, the use of nephrectomy in mRCC has remained substantially stable for the last decades. More than 85% of patients included in randomized trials and expanded access programs published from 2003 to 2019 had undergone previous nephrectomy [21], which means that current evidence driving the clinical practice, originates from a nephrectomized population and supports the use of CN also in the metastatic setting.

More recently, the phase II GETUG-AFU-26 NIVOREN trial explored the impact of nivolumab in 111 patients, mainly with intermediate (45%) and poor (49%) IMDC risk, who did not undergo upfront CN [22]. A lower mPFS, mOS, and ORR (of 2.7 months, 15.9 months and 16%, respectively) was observed in those patients than expected from the Check-Mate 025 study results [3, 22]. Moreover, among patients with an evaluable primary renal tumor, only 6% experienced shrinkage of more than 30% [22]. In a meta-analysis investigating the efficacy of first-line ICI combination therapies compared to single-agent VEGFR-TKI sunitinib in mRCC patients with and without previous CN, the benefit of immunotherapy combinations seemed not to differ between those two subgroups [19].

Our findings confirm the favourable impact of nephrectomy on the clinical outcome of patients who had failed VEGFR-TKIs for mRCC and received single-agent nivolumab in subsequent lines, similarly to what a previous analysis reported in the same setting [23]. More interestingly, the benefit of the previous nephrectomy was evident for patients with a better prognosis according to the Meet-URO score, belonging to groups 1 to 3. In those patients, the nephrectomy was associated with a significant 60% reduction in the risk of death. Conversely, in patients classified as Meet-URO score 4 and 5 the previous nephrectomy did not have an impact on OS. These results align with previous data reported with TKIs, indicating a lack of benefit from CN in patients with more than three IMDC risk factors [16]. Hence, the Meet-URO score confirms the lack of benefit of the previous nephrectomy in IMDC poor-risk patients (which are included in the Meet-URO score groups 4 and 5) and also identifies lack of benefit in a subgroup of IMDC intermediate-risk patients defined as group 4 by the Meet-URO score [6].

Taking together those observations confirmed the prognostic positive role of nephrectomy which appears to confer a favorable outcome to mRCC patients. However, without being able to ascertain the predictive value in terms of tumor response to systemic treatments in the metastatic setting still remains undefined. The possible predictive role of this surgical procedure in terms of response to specific systemic treatments, in the metastatic setting.

Limitations of the present study are the retrospective design, the undefined intent of the previous nephrectomy (i.e. cytoreductive vs curative), the relatively small number of patients in the no-nephrectomy group and potential positive selection bias (as the study included patients who were able to receive treatments beyond first-line VEGFR-TKIs). A further limitation might be the applicability to the first-line setting, as increasing first-line ICI combinations will reduce the percentage of patients who will be offered second-line immunotherapy. However, it should also be noted that a small proportion of IMDC good-risk patients will likely continue to receive a TKI-nivolumab therapeutic sequence [24].

Nevertheless, we believe that a more accurate prognostic stratification might help to identify mRCC patients who would likely benefit from nephrectomy and deserves further prospective analyses by treatment setting.

## Conclusions

Our analysis showed that prior nephrectomy has a generally favorable effect on the prognosis of pretreated mRCC patients receiving immunotherapy. In particular, for patients with Meet-URO scores of 1 to 3, who are the only ones benefiting from prior nephrectomy, more research into the therapeutic value of cytoreductive nephrectomy, appropriate scheduling and patient selection is warranted. The IMDC poor-risk patients (including the Meet-URO score groups 4 and 5) are confirmed not to benefit from the absence of the primitive tumor for prior nephrectomy, but also a subgroup of IMDC intermediate-risk patients defined as group 4 by the Meet-URO score do not appear to receive this advantage, either.

## Supplementary Information

**Additional file 1: Figure S1. **Stratification of patients by IMDC score at disease onset and nivolumab treatment start (N = 493)*. * Missing data for 63 patients. Abbrevviations: PG prognostic group, RG risk group.

**Additional file 2: Figure S2. **Stratification of patients by IMDC and Meet-URO scores at nivolumab treatment start (N = 556)*. * Treatment line / patients: 2nd/384, 3rd/118, 4th/41, 5th/11, 6th/1, 7th/1. Abbreviations: PG prognostic group, RG risk group.

**Additional file 3: Figure S3. **Kaplan Meiers curves showing the prognostic role of nephrectomy in mRCC patients according to the type of nephrectomy.

## Data Availability

The datasets used and/or analysed during the current study are available from the corresponding author on reasonable request.
