# Supplementary figures and images for: The prognostic value of the previous nephrectomy in pretreated metastatic renal cell carcinoma receiving immunotherapy: a sub-analysis of the Meet-URO 15 study
Source: J Transl Med. 2022 Sep 30;20:435. doi: 10.1186/s12967-022-03601-6 (PMC9524042; doi:10.1186/s12967-022-03601-6)

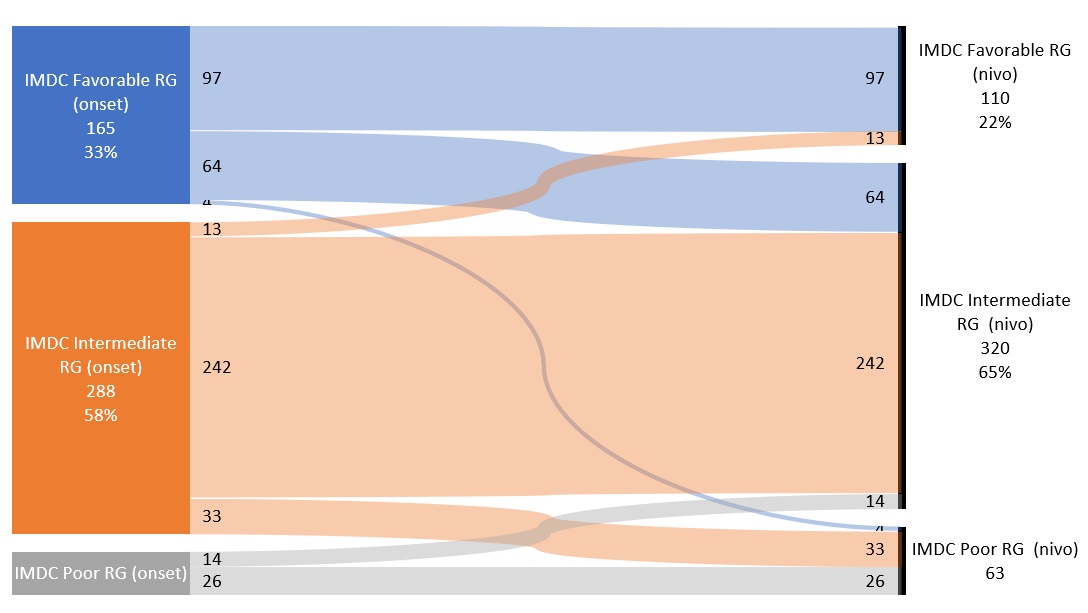

Supplement: Supplementary file 1 — Additional file 1: Figure S1. Stratification of patients by IMDC score at disease onset and nivolumab treatment start (N = 493)*. * Missing data for 63 patients. Abbrevviations: PG prognostic group, RG risk group. [file 12967_2022_3601_MOESM1_ESM.jpg]

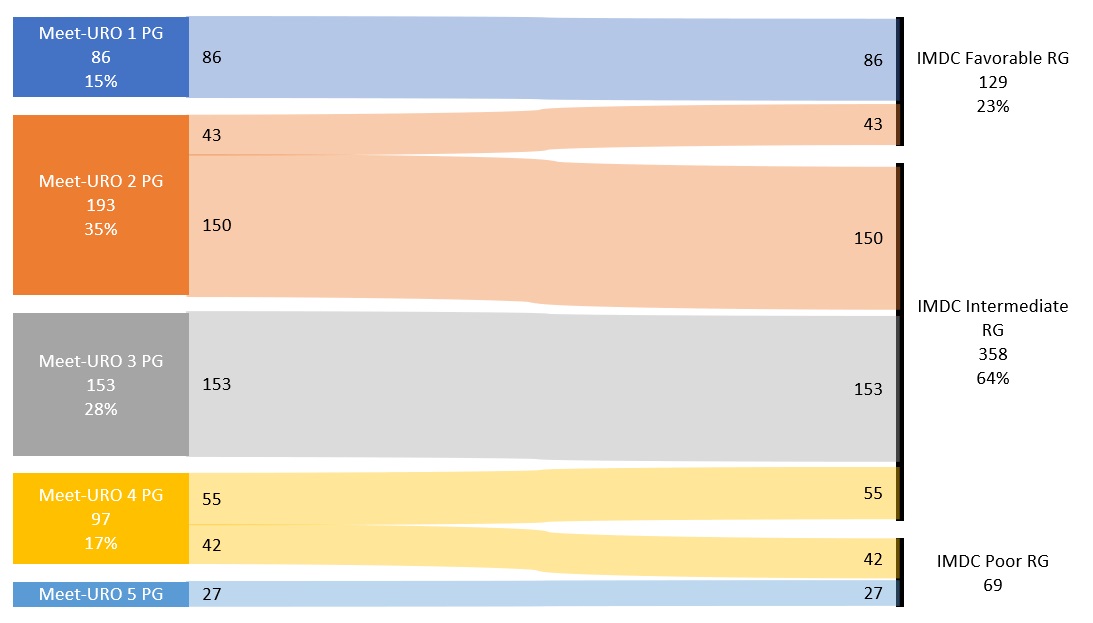

Supplement: Supplementary file 2 — Additional file 2: Figure S2. Stratification of patients by IMDC and Meet-URO scores at nivolumab treatment start (N = 556)*. * Treatment line / patients: 2nd/384, 3rd/118, 4th/41, 5th/11, 6th/1, 7th/1. Abbreviations: PG prognostic group, RG risk group. [file 12967_2022_3601_MOESM2_ESM.jpg]

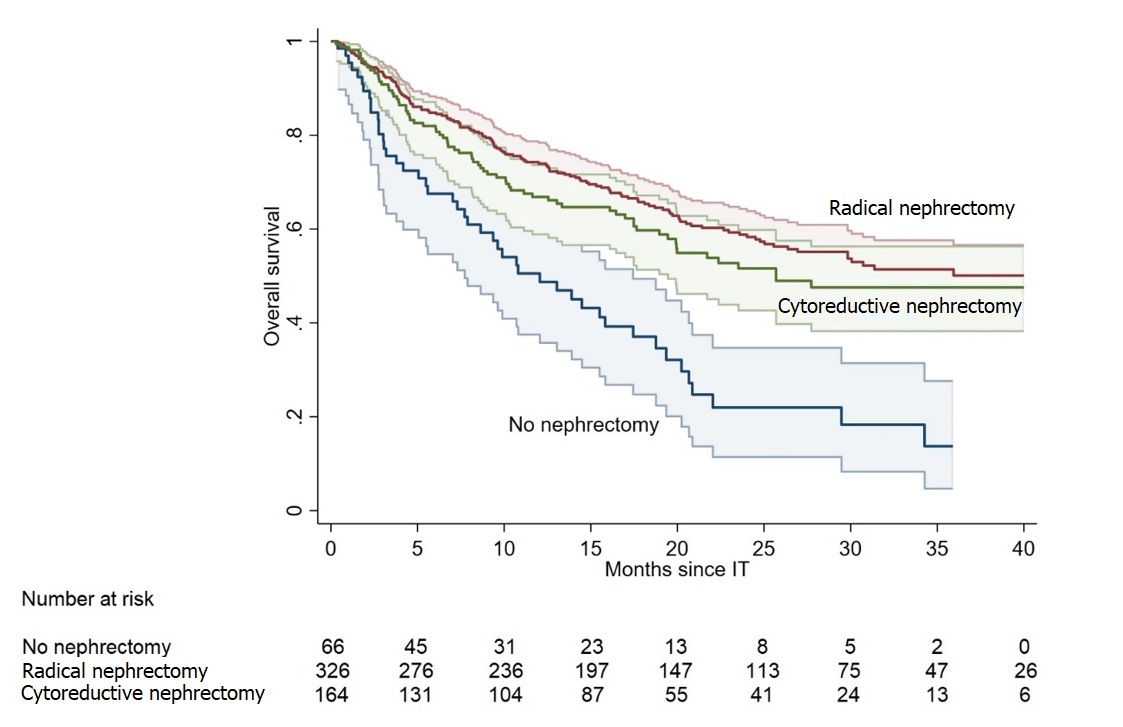

Supplement: Supplementary file 3 — Additional file 3: Figure S3. Kaplan Meiers curves showing the prognostic role of nephrectomy in mRCC patients according to the type of nephrectomy. [file 12967_2022_3601_MOESM3_ESM.jpg]
